# Minimal Intervention Dentistry (MID) mainstream or unconventional option? Study exploring the impact of COVID-19 on paediatric dentists’ views and practices of MID for managing carious primary teeth in children across the United Kingdom and European Union

**DOI:** 10.1007/s40368-022-00746-2

**Published:** 2022-10-31

**Authors:** A. BaniHani, A. Hamid, J. Van Eeckhoven, S. Gizani, S. Albadri

**Affiliations:** 1 Department of Paediatric Dentistry, School of Dentistry, University of Leeds, Worsley Building, The Clarendon Way, Leeds, LS2 9LU UK; 2 Bristol Dental Hospital, Bristol, UK; 3 School of Biology, University of Leeds, Leeds, UK; 4 Department of Paediatric Dentistry, National and Kapodistrian University of Athens, Athens, Greece; 5 School of Dentistry, University of Liverpool, Liverpool, UK

**Keywords:** COVID-19, Minimal intervention dentistry, Hall technique, Silver diamine fluoride, Dental caries, Primary teeth, Paediatric dentists, Oral healthcare for children

## Abstract

**Purpose:**

To explore the techniques used to manage carious primary teeth during the COVID-19 pandemic by paediatric dentists and dentists with a special interest in paediatric dentistry (DwSI) who are members of the British Society of Paediatric Dentistry (BSPD) and the European Academy of Paediatric Dentistry (EAPD), and their views on the use of minimal intervention dentistry (MID) in children prior to, during and post the COVID era.

**Methods:**

A total of 212 paediatric dentists and DwSI completed an online questionnaire. Six MID techniques were explored: fissure sealants, resin infiltration, Hall Technique (HT), 38% silver diamine fluoride (SDF), atraumatic restorative treatment (ART), stepwise removal and selective caries removal.

**Results:**

The majority were specialists (26%) followed by clinical academics (23.1%) working mainly in university teaching hospitals (46.2%). Routine dental treatment for children with carious primary teeth was provided by the majority (92.5%) during the pandemic. HT (96%) and 38% SDF (65.7%) were the most commonly used techniques among the BSPD members whereas conventional restoration of non-selective caries removal and pulp therapy remained the most widely used technique among the EAPD members (66.2%). Most of the MID techniques were used as a treatment option (48.1%) rather than a choice (43.4%), with most of these choices having been affected by the patient’s behaviour (82.5%). More than one thirds (39.2%) of the participants were reluctant to adopt MID after the pandemic. Several barriers such as lack of teaching and confidence as well as perceived lack of evidence were identified.

**Conclusion:**

A range of MID techniques is practiced broadly by a sample of paediatric dentists and DwSI across the United Kingdom (U.K) and European Union (E.U). The majority of clinicians are willing to continue using these techniques going forward after COVID restrictions are lifted. The pandemic served as an opportunity for many dentists to become familiar with various MID practices, such as SDF, which has been already established some time ago.

## Background

The COVID-19 pandemic is an ongoing global crisis caused by Severe Acute Respiratory Syndrome Coronavirus 2 (SARS-CoV-2). Children under the age of 16 make up around 2% of total COVID-19 cases worldwide and are mainly asymptomatic, this combined with the long incubation period (up to 14 days) means children can contribute significantly to the transmission of the disease (RCPCH 2020). The unprecedented nature of the pandemic has significantly disrupted the provision of dental care. In March 2020, as a response to the COVID-19 outbreak, governments worldwide introduced national lockdowns to minimise the transmission of the virus. Due to the risks of COVID-19 in the dental setting routine dental treatment, including all aerosol-generating procedures (AGPs) were suspended, necessitating the need for alternative caries management.

MID has developed based on biological concepts with the aim to alter the environment of carious lesion preventing progression by isolating them from the cariogenic biofilm. It covers a spectrum of child-friendly techniques, ranging from no carious tissue removal to selective carious tissue removal, preserving as much of the tooth structure as possible (Innes et al. 2016; BaniHani et al. 2019, 2021; Hussein et al. 2020). Several MID techniques provide a safe, low-risk aerosol-generating procedures (LRAGPs) with high-quality treatment approaches that are highly accepted by children (Al-Halabi et al. 2020; Banihani et al. 2020). Thus considered an appropriate management technique during and after the COVID-19 era. These techniques involve sealants (fissure sealants and resin infiltration), topical application of 38% SDF, the HT, ART and selective removal of carious tissue (Innes et al. 2016; Al-Halabi et al. 2020; BaniHani et al. 2020; BaniHani et al. 2021).

Fissure sealants aim to seal and inhibit the further progression of carious lesions by isolating caries from the surface biofilm, thus delaying or preventing the need for AGPs (Naaman et al. 2017). Unlike fissure sealants, resin infiltration creates a diffusion barrier within the carious lesion by filling and reinforcing the demineralised lesion with a low-viscosity resin (Doméjean et al. 2015).

SDF is a topical colourless ammonia liquid containing silver and fluoride. Silver is antibacterial, and it acts synergistically with fluoride, which is known to enhance the remineralisation of dental hard tissue, arrest dental caries and prevent new lesions from forming on remaining tooth surfaces (Seifo et al. 2020). The literature supports 38% SDF as the optimal concentration to arrest dental caries (Tolba et al. 2019; BaniHani et al. 2021). The main drawback of SDF is that it stains carious teeth black, thus thorough discussion with parents prior to its use is paramount especially if SDF is to be applied to carious primary anterior teeth (Seifo et al. 2020).

Unlike the invasive conventional preformed metal crowns (PMCs), HT aims to seal carious lesion from sugary substrates by means of PMCs without local anaesthetic, caries removal or any tooth preparation (Innes et al. 2006). As a response, carious lesion composition will be shifted towards less cariogenic flora, arresting or slowing down caries progression as a result, and protecting primary molars until shedding (Hussein et al. 2020). The technique is considered quick and easy to use where an appropriately sized PMC would be chosen and filled with glass ionomer cement before being fitted over the carious primary molar by either the dentist’s finger pressure or the child’s biting force (Innes et al. 2006). Elastomeric orthodontic separators are usually placed prior to placing Hall PMCs where tight contact is present between primary molars to create space mesially and distally to the tooth.

ART is another MID technique that was first introduced in developing countries to increase children’s access to dental treatment due to the lack of facilities or accessibility to the dental clinic. It involves preventive and restorative measures in which caries is removed usually using hand instruments without local anaesthesia, and the intact fissures are sealed with High Viscosity Glass Ionomer Cement (HVGIC) (Innes et al. 2016; de Amorim et al. 2018). Moreover, selective removal of carious tissue, known as partial or incomplete caries removal, is mainly indicated in deep cavitated lesions where caries is extending to the pulpal third to avoid pulp exposure and stress to the pulp. It includes selective removal of carious tissue pulpally until either soft dentine, where caries is easily scooped up with little force being required, is reached or firm dentine, which is resistant to hand excavator, is reached. Periphery of the cavity should be cleaned to hard dentine that is similar to sound dentine to allow a tight seal and placement of a durable restoration (Innes et al. 2016; BaniHani et al. 2018a, b).

In response to the high proportion of dental caries among children worldwide and its profound consequences including pain, infection and negative impact on quality of life, combined with the limited access to AGPs and dental general anaesthetic lists, MID in caries management has been adopted by some paediatric dentists during the COVID-19 pandemic as an alternative to the conventional approach of non-selective caries removal and pulp therapy using high-speed handpiece, and 3-in-1 syringe (BaniHani et al. 2020).

The concept of MID was introduced in the early 1990s and has been gaining popularity ever since (Dawson and Mackinson 1992). The number of studies, specifically systematic and umbrella reviews, evaluating the effectiveness of different MID techniques for caries management in children has significantly increased in recent years (BaniHani et al. 2021). Consequently, paediatric dentists have routinely been using several MID techniques prior to the COVID-19 pandemic.

Some emerging studies focusing on the oral health of paediatric patients during, and post pandemic, have unsurprisingly reported an increase in the proportion of patients presenting with dental emergencies during the pandemic, who have been managed with extractions or pulp therapy (Fux-Noy et al. 2021).

There remain few studies understanding paediatric dentists’ practices in caries management of primary teeth during the COVID-19 pandemic, and even fewer exploring their views on the use of MID for the management of dental caries in children during and after the COVID pandemic. Only one study, conducted in Jeddah, supported our hypothesis and found the use of these MID techniques increased throughout the profession during the COVID pandemic (Alamoudi et al. 2022). Perhaps, as a result of the various resources being launched raised awareness of MID, for example, the British Society of Paediatric Dentistry (BSPD) released resources to support the use of SDF in arresting dental caries in primary teeth in 2020 (BSPD 2020).

Several authors have recommended the continuation of MID techniques post-pandemic, which may help reduce any anxiety regarding COVID-19 amongst paediatric patients, carers and healthcare providers (Al Halabi et al. 2020; Alamoudi et al. 2022).

Therefore, as more evidence emerges this study aims to explore how paediatric dentists are managing carious primary teeth during the pandemic, from the start of the first lockdown in March 2020 to the time of conducting the study in July 2021, and their views on the use of MID for the management of carious primary teeth in children during and post the COVID era.

## Materials and methods

### Participants and ethics

This study involved a questionnaire-based online survey, using OnlineSurveys.NET (https://www.onlinesurveys.ac.uk). The online questionnaire was sent to all members of the BSPD and the EAPD. Members of the BSPD and EAPD were approached by the secretaries of both dental organisations to participate in the study by email in April 2021. Participants who were members of both EAPD and BSPD were asked to complete the questionnaire once only.

Approval was obtained from the Dental Research Ethics Committee (DREC), University of Leeds (reference number 021120/ABH/310).

Participants were included in the study if they met the following inclusion criteria:

- Paediatric dentists including specialist trainees, postgraduate students, clinical academics, specialists and consultants with no limited number of years in their membership period length.
- DwSI in paediatric dentistry where more than 50% of their dental practice capacity is dedicated to treat children under the age of 10 years.
- Dentists speak the English language.

A cover letter was sent with the survey, explaining the aim of the study and inclusion criteria. Implied consent was obtained from all participants prior to the completion of the survey. A reminder email was sent out to all potential participants 3 weeks after the initial contact. The survey was sent out again two months later to increase the response rate.

Pre-pandemic era in the current study was referred to as the time prior to March 2021. Whereas the COVID pandemic period included the time from the start of the first lockdown in March 2020 in the U.K and E.U to the time of conducting the study in July 2021 where COVID-19 restrictions were still uneased in the U.K and most of the E.U countries. Post-COVID-era referred to the period when all the restrictions related to the pandemic are lifted with no set date that could be determined in the study due to the ongoing pandemic.

### The questionnaire

The online questionnaire was developed by the research team and it comprised three sections with a total of 20 multiple-choice questions. The majority of the questions included free space for comments to give a deeper understanding of some of the situations. The questionnaire aimed to assess the following:

- Demographics (6 questions), including the country and clinical setting respondents work in, current job role, and number of years of experience they had been practising paediatric dentistry.
- Current techniques used to manage carious primary teeth during the pandemic (2 questions).
- Participants views’ on different MID techniques and their likability to adopt MID for managing carious primary teeth during and post the COVID era (12 questions), including questions on how often these techniques were used before the pandemic when they were used, reasons for using them, and whether COVID-19 has changed their beliefs about using MID.

Six MID techniques were explored in the current study; sealants including both fissure sealants and resin infiltration, HT, 38% SDF, ART, stepwise removal and selective caries removal.

The questionnaire was based on previously published, ethically approved and validated questions (Roberts et al. 2018), however, some questions were modified and others were created based on the literature and national guidelines. The questionnaire was piloted prior to its use among a group of trainees, specialist, and consultants in paediatric dentistry (*N* = 10) at the University of Leeds, and the questionnaire was modified according to their feedback.

### Sample size calculation

After seeking statistician advice, the questionnaire was sent to all paediatric dentists and DwSI in paediatric dentistry who are members of the BSPD and EAPD dental societies. No power calculation could be carried out as no previous studies were published on this topic.

### Statistical analysis

Descriptive data analysis was carried out using the SelectSurvey.NET. Thematic analysis was used to analyse open questions. In addition, a generalised linear model (binomial error with logit link function) and a log-likelihood ratio test were used to investigate the effect of different demographic factors (country of training, clinical setting of current place of work and years of practicing paediatric dentistry) on the usage of MID among participants, using R (v4.1.3).

## Results

In total, 212 participants completed the survey; 70 were members of the BSPD and 142 were members of the EAPD.

### Demographics

The demographics of the participants are summarised in Table 1. The majority (48.1%) of the participants worked in the E.U at the time of the survey, followed by the U.K (35.8%). Only 16% worked in non-E.U countries mainly Turkey (28.1%) and the United States of America (12.5%).

**Table 1:** Summarises the demographics of the participants from both dental societies; BSPD (*N* = 70) and EAPD (*N* = 142)

Variable	BSPD participants *N* (70)	EAPD participants *N* (142)	Total *N* (212)
*Country of work*			
UK	68 (97.2%)	8 (5.6%)	76 (35.8%)
EU	1 (1.4%)	101 (71.2%)	102 (48.1%)
Non-EU	1 (1.4%)	33 (23.2%)	34 (16%)
*Current job*			
Dentists with special interest in paediatric dentistry	19 (27.1%)	15 (10.6%)	34 (16%)
Dentist completing specialist training	6 (8.6%)	16 (11.3%)	22 (10.4%)
Postgraduate student	6 (8.6%)	9 (6.3%)	15 (7%)
Clinical academic	6 (8.6%)	43 (30.3%)	49 (23.1%)
Specialist	17 (24.3%)	38 (26.8%)	55 (26%)
Consultant	16 (22.8%)	21 (14.7%)	37 (17.5%)
*Country of training in paediatric dentistry*			
UK	50 (98%)	10 (7.8%)	60 (33.6%)
EU	0.0	83 (65.4%)	83 (46.6%)
Non-EU	1 (2%)	34 (26.8)	35 (19.8%)
*Clinical setting of current place of work*			
University teaching hospital	32 (45.7%)	66 (46.5%)	98 (46.2%)
Community-based hospitals	32 (45.7%)	19 (13.4%)	51 (24%)
Private practice	3 (4.3%)	41 (28.8%)	44 (20.9%)
Mixed	3 (4.3%)	16 (11.3%)	19 (8.9%)
*Years of practicing paediatric dentistry*			
Less than 5 years	18 (25.7%)	28 (19.7%)	46 (21.7%)
5–10 years	8 (11.4%)	25 (17.6%)	33 (15.5%)
10–15 years	19 (27.1%)	26 (18.3%)	45 (21.2%)
More than 15 years	25 (35.8%)	63 (44.4%)	88 (41.6%)
*Involvement in teaching*			
Yes	56 (80%)	106 (74.6%	162 (76.4%)
No	14 (20%)	36 (25.4%)	50 (23.6%)

Less than a third of participants were working as specialists in paediatric dentistry (26%), another 17.5% were consultants in paediatric dentistry.

DwSI in paediatric dentistry made up 16% of all responses, 23.1% of participants were clinical academics, another 17.4% were dentists completing specialist training or postgraduate students. The majority of the participants who have completed their training in paediatric dentistry had their training in E.U (46.6%) followed by U.K (33.6%).

Less than half (46.2%) worked in dental teaching hospitals, 24% in the community dental service, 20.9% in private practice and 8.9% worked in a mixed setting. More than half (65.8%) have been practicing paediatric dentistry for greater than 10 years with over three quarters (76.4%) involved with teaching or training other dentists/dental care professionals.

### Current techniques used to manage carious primary teeth during the pandemic

During the COVID-19 pandemic, from the start of the first lockdown in March 2020 to the time of conducting the study in July 2021, routine dental treatment to children with carious primary teeth was provided by the majority (92.5%) of the participants of both dental societies, BSPD (88.6%) and EAPD (94.4%).

**Fig. 1:**
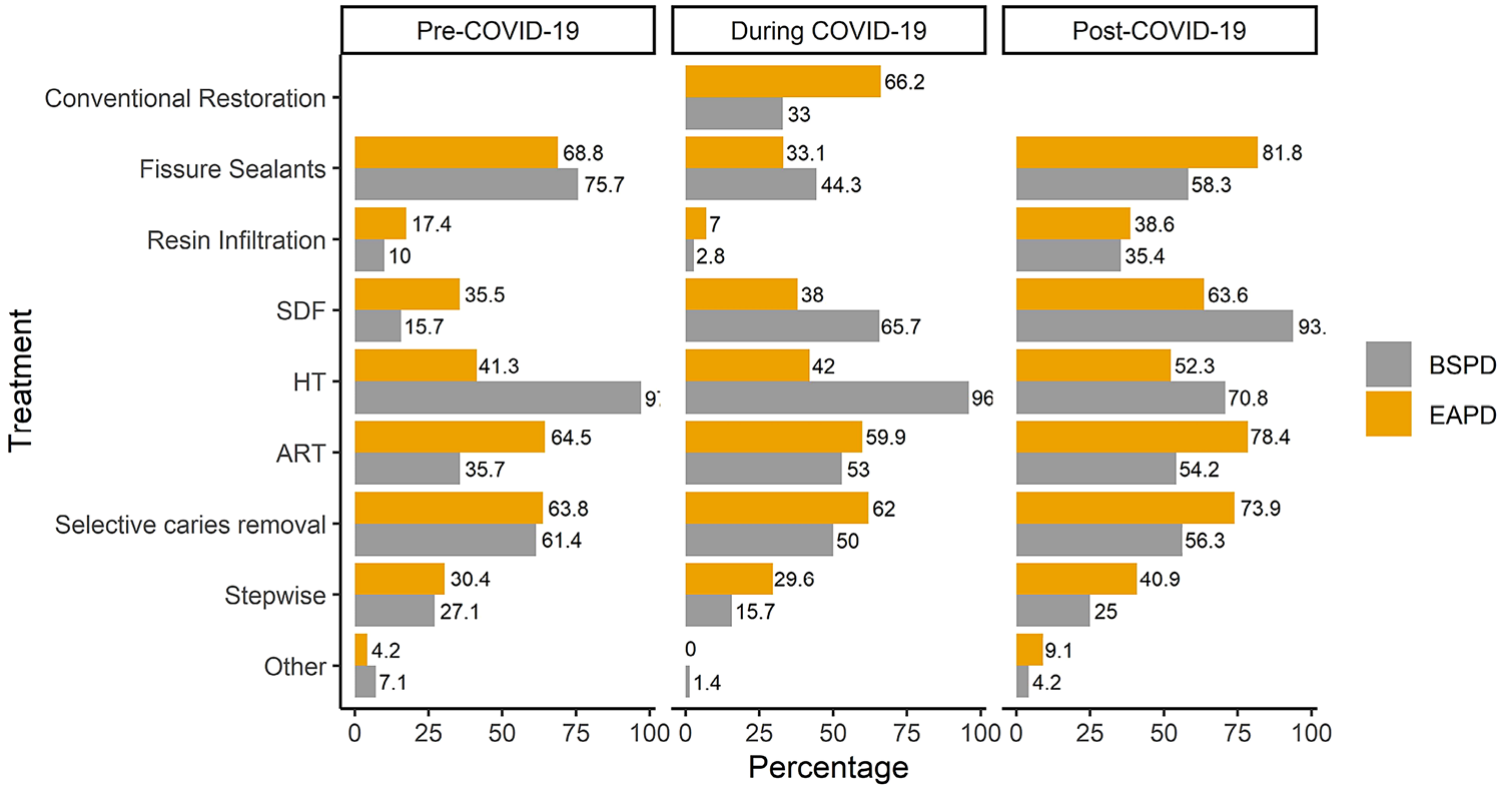
The most commonly used techniques used by the participants for the management of carious primary teeth in children prior to, during and post the COVID-19 pandemic. *The use of conventional restoration prior to and post the COVID-19 pandemic was not assessed among the participants in the current study

With regards to the management of carious primary teeth during the pandemic, the following techniques were most used by the participants of the BSPD; HT (96%), topical application of 38% SDF (65.7%), ART (53%), selective caries removal (50%), fissure sealants (44.3%), conventional restoration of non-selective caries removal and pulp therapy (33%), stepwise excavation (15.7%), resin infiltration (2.8%), and teeth extraction (1.4%). For the EAPD participants, the most common techniques used were the conventional restoration of non-selective caries removal and pulp therapy (66.2%), selective caries removal (62%), ART (59.9%), HT (42%), topical application of 38% SDF (38%), fissure sealants (33.1%), stepwise excavation (29.6%), and resin infiltration (7%). Figure 1 summarises the management techniques used for carious primary teeth during the COVID-19 pandemic by the participants.

### Participants views on the use of MID prior to the pandemic

Table 2 summarises participants views on using MID for the management of carious primary teeth prior to the pandemic.

**Table 2:** Participants views on using MID techniques for the management of carious primary teeth prior to COVID-19 pandemic including the reasons for using MID techniques

Variable	BSPD participants *N* (70)	EAPD participants *N* (142)	Total *N* (212)
*Did you use MID prior to the pandemic*			
Yes	62 (88.6%)	134 (94.4%)	196 (92.5%)
No	8 (11.4%)	8 (5.6%)	16 (7.5%)
*How often did you use MID*			
Never	0.0	0.0	0.0
Occasionally (less than 50% of all cases)	19 (27.1%)	83 (58.5%)	102 (48.1%)
Often (between 50 and 75% of all cases)	42 (60%)	50 (35.2%)	92 (43.4%)
Always	9 (12.9%)	9 (6.3%)	18 (8.5%)
*When MID was used*			
As treatment option	33 (47.1%)	54 (38%	87 (41%)
As treatment choice	29 (41.4%)	44 (31%)	73 (34.5%)
When conventional restoration could not be provided	8 (11.5%)	44 (31%)	52 (24.5%)
*Reasons for using MID prior the pandemic*			
Atraumatic techniques where no local anaesthetic required	51 (72.9%)	89 (63.6%)	140 (66%)
Maintain tooth structure and reduce risk of pulp exposure	59 (84.3%)	106 (75.7%)	165 (77.8%)
Easy to do	41 (58.6%)	55 (39.3%)	122 (57.5%)
Short procedure	43 (61.4%)	79 (56.4%)	122 (57.5%)
Highly accepted by children	61 (87.1%	91 (65%)	152 (71.7%)
Appropriate for anxious children	61 (87.1%	107 (76.4%)	168 (79.2%)
Appropriate for young children	57 (81.4%)	101 (72.1%)	158 (74.5%)
Strong evidence of the clinical effectiveness of MID	59 (84.3%)	78 (55.7%)	137 (64.6%)
*Was your choice of using MID affected by the patient behaviour*			
Yes	55 (78.6%)	120 (84.5%)	175 (82.5%)
No	15 (21.4%)	22 (15.5%)	37 (17.5%)

Prior to the COVID-19 pandemic, more than two-thirds (72.6%) of the BSPD participants and less than half of the EAPD participants (41.5%) reported using MID techniques ‘always’ or ‘often’, accounting for over 50% of all management techniques provided for carious primary teeth.

Figure 1 summarises the management techniques used for carious primary teeth prior to the COVID-19 pandemic by the participants. The most common techniques used prior to the pandemic by BSPD participants were: HT (97.1%), fissure sealants (75.7%), selective caries removal (61.4%), ART (35.7%), stepwise excavation (27.1%), topical application of 38% SDF (15.7%), resin infiltration (10%), prevention and carious lesion monitoring including topical application of 5% fluoride varnish (5.7%), and silver modified ART (1.4%). The majority (47.1%) of these techniques were used as a treatment option for carious primary teeth, 41.4% were a treatment of choice, and only 11.5% were used only if conventional restoration was not possible.

For the EAPD participants, the figures were as follow: fissure sealants (68.8%), ART (64.5%), selective caries removal (63.8%), HT (41.3%), topical application of 38% SDF (35.5%), stepwise excavation (30.4%), resin infiltration (17.4%), prevention and carious lesion monitoring including topical application of 5% fluoride varnish application (3.5%), and non-restorative caries control (0.7%). The majority of these techniques were used as a treatment option for carious primary teeth (38%), with only 31% used as a treatment of choice, or when the conventional restoration was not possible.

Among the reasons listed for using MID techniques before the COVID-19 pandemic participants gave the following responses: appropriate management technique for anxious children (79.2%), to maintain tooth structure and reduce risk of pulp exposure (77.8%), appropriate management technique for young children (74.5%), highly accepted by children (71.7%), and atraumatic techniques where no local anaesthetic required prior to treatment (66%).

In addition, the majority of the participants believed that their choice of using MID was affected by the child`s behaviour and cooperation (78.6% and 84.5% of the BSPD and EAPD participants, respectively).

Approximately one-third (37.7%) of all participants believe the COVID-19 pandemic has changed their views and practices of MID (51.4% of the BSPD participants, 31% of the EAPD participants). Years spent practicing paediatric dentistry was the only demographic factor found to have a statistically significant effect on whether COVID-19 has changed participants view of MID (*p* = 0.010). Practitioners that have practiced for over 15 years were found to be less inclined to use MID techniques post-COVID-19.

### Thematic analysis of the open questions

Responses to open questions by participants of both societies revealed one common theme; “benefits related to the use of MID” which highlighted the advantage of using MID in managing carious primary teeth thus reducing the need for AGPs during the pandemic as well as patient waiting lists for dental treatment, both in the chair and under GA. The following comments were left by some participants:

“*Planned to use MID to reduce both the number of AGPs and the length of AGP appointments” BSPD member, U.K.*

“*The pandemic has made me consider MID as a more acceptable short/medium term solution as a first line, rather than using it as a last resort” EAPD member, Netherlands.*

*“I always believed in MID techniques and have advocated for them. I have never seen the benefit of getting sufficient cooperation for local anaesthesia to do a filling when a Hall PMCs could be placed much easier and is more effective! I think COVID has helped highlight how effective sealing in caries (HT, fissure sealants) and high level prevention is” BSPD member, U.K.*

Additional comments left by the BSPD participants have highlighted a unique theme concerning the introduction of 38% SDF for the management of carious primary teeth during the pandemic, reported by more than half of the participants (55.5%). Comments on this theme included the following:

*“ I was already interested in MID, I believe in HT and procedures that preserve the vitality of the pulp (eg stepwise/selective caries removal). COVID 19 has reinforced further my belief in the value of these techniques. I am trying to introduce & develop the use of SDF within my service at the moment” BSPD member, U.K.*

* “I have started using SDF since the pandemic started” BSPD member, U.K.*

*“I would now add SDF as a treatment option for carious primary teeth” BSPD member, U.K.*

### Participant’s likelihood to adopt MID post the COVID-19 era

Participants views on using MID techniques post-COVID-19 era are summarised in Table 3.

**Table 3:** Participants views on using MID techniques for the management of carious primary teeth after the COVID-19 pandemic

Variable	BSPD participants *N* (70)	EAPD participants *N* (142)	Total *N* (212)
*Did COVID-19 change your believes about MID?*			
Yes	36 (51.4%)	45 (31%)	80 (37.7%)
No	34 (48.6%)	97 (69%)	132 (62.3%)
*Do you feel more inclined to use MID techniques after pandemic*			
Yes	51 (72.9%)	78 (55%)	129 (60.8%)
No	19 (27.1%)	64 (45%)	83 (39.2%)
*If no, specify a reason*			
No enough teaching with further training/teaching required	2 (9.1%)	16 (25.4%)	18 (8.5%)
Insufficient evidence available on its efficacy	1 (4.5%)	12 (19%)	13 (6.1%)
Lack of enough confidence			
Difficulty in obtaining dental materials required for MID	0.0	12 (19%)	12 (5.7%)
Patient choice	0.0	4 (6.3%)	4 (1.9%)
*If yes, specify a reason*			
They are considered LRAGPs	44 (80%)	73 (67%)	117 (55.2%)
Can be completed in a short period of time	28 (50.9%)	76 (69.7%)	104 (49%)
Easy to do	30 (54.5%)	58 (53.2%)	88 (41.5%)
Less challenging for children compared to conventional restoration	44 (80%)	80 (73.4%)	124 (58.5%)
Emerging evidence supporting the use of MID during the pandemic	41 (74.5%)	46 (42.2%)	87 (41%)
*If you are using MID during the pandemic, will you continue using MID after the pandemic*			
Yes	70 (100%)	137 (96.5%)	207 (97.6%)
No	0.0	5 (3.5%)	5 (2.4%)
*Are you willing to teach MID to dental students and other dental care professionals*			
Yes	70 (100%)	125 (88.7%)	195 (91.9%)
No	0.0	17 (11.3%)	17 (8.1%)

Only 27.1% and 45% of the BSPD and EAPD participants, respectively, stated that they do not feel more inclined to use MID after the COVID-19 pandemic. Reasons stated for this were mainly due to not receiving enough teaching with further training/teaching required (9.1% BSPD participants, 25.4% EAPD participants), insufficient evidence available on its efficacy (4.5% BSPD participants, 19% EAPD participants), lack of enough confidence (19% EAPD participants), difficulty in obtaining dental materials required for MID (6.3% of EAPD participants), and patient choice (9.1% BSPD participants, 11.1% EAPD participants).

For those who feel more inclined to use MID after the pandemic, topical application of 38% SDF (93.8%) and HT (70.8%) were the two most popular techniques to be considered by BSPD participants. Whereas, amongst EAPD members, fissure sealants (81.8%) and ART (78.4%) remained the most common techniques to be considered (Fig. 1).

Reasons for considering MID after the pandemic included the following: less challenging techniques for children compared to the conventional approach of non-selective caries removal and pulp therapy (58.5%), considered decreased/ non-AGPs (55.2%), can be completed in a short period of time (49%), easy to do (41.5%) as well as increased emerging evidence supporting the use of MID during the COVID-19 pandemic (41%). In addition, the vast majority of participants were willing to teach MID techniques to dental students and other dental care professionals (100% BSPD participants, 91.1% EAPD participants).

## Discussion

To the best of our knowledge,e this is the first study to explore the various MID techniques used by paediatric dentists and DwSI in paediatric dentistry pre and during the COVID-19 pandemic in the U.K and E.U. This study also explores clinician’s views, perceptions and experiences of MID pre, during and post-pandemic.

The majority of the respondents worked across the whole of U.K and E.U, and worked in a mixture of settings (predominantly teaching hospitals, community clinics which is where most specialists work and private practice), suggesting that respondents were a representative sample. The majority of the respondents from the BSPD were practicing in the U.K (97%) whereas most of the respondents from the EAPD were practicing in E.U (71.2%).

During the pandemic, various MID techniques were used by the respondents for the management of carious primary teeth in children. The results suggested that HT and topical application of 38% SDF were the most commonly used techniques and were commonly favoured over the conventional restoration of non-selective caries removal and pulp therapy among the paediatric dentists and DwSI in the U.K. Whereas in E.U, the conventional restoration of non-selective caries removal and pulp therapy remained the most widely used technique, despite these techniques carrying a risk of transmitting and spreading the infection to patients, dental staff and the publics as AGPs (BaniHani et al. 2020). The latter could be explained by the fact that the uncertainty about the duration of restrictions imposed on the provision of routine dental treatment by the governments during the lockdown may have led the dentists practicing in the E.U to more radical treatments such as complete caries removal and pulp therapy. In addition, the increased use of pulp therapy could have reflected the severity of the dental condition, for example, acute pulpitis and acute apical periodontitis, that led the patients to seek help.

HT and SDF did not seem practiced much amongst clinicians in the E.U prior to and during the pandemic despite the strong supportive evidence of these techniques. Surprisingly the majority of the EAPD participants were clinical academics (30.3%) (Innes et al. 2015; Gao et al. 2016; BaniHani et al. 2018a, b). With the exception of the Netherlands, where MID is the treatment of choice according to the new clinical guidelines. HT showed a success rate of over 90% in several studies (Innes et al. 2007; Innes et al. 2015; BaniHani et al. 2018a, b), and was found to be as successful and more cost-effective than the conventional restoration of non-selective caries removal and pulp therapy (BaniHani et al. 2019). Whereas, topical application of 38% SDF had an overall caries arrest rate of 81% in children (Gao et al. 2016). In addition, a recent published umbrella review aimed to appraise the evidence behind the use of several MID techniques for managing dentinal carious lesions in primary teeth. This concluded that MID techniques, namely 38% SDF, HT, selective removal of carious tissue, and ART for a single surface cavity, appear to be effective in arresting the progress of dentinal caries in primary teeth when compared to no treatment, or conventional restorations (BaniHani et al. 2021). Concerning the SDF and HT, the review concluded that 38% SDF has a significant caries arrest effect in primary teeth (*p* < 0.05), and its success rate in arresting dental caries increased when it was applied twice (range between 53 and 91%) rather than once a year (range between 31 and 79%). Moreover, PMCs placed using the HT were likely to reduce discomfort at the time of treatment, the risk of major failure (pulp treatment or extraction needed) and pain compared to conventional restorations.

Prior to the pandemic, it is interesting to note that a significant number of respondents used MID always or often for the management of carious primary teeth, highlighting the fact that paediatric dentists are familiar with MID and the evidence base supporting its use. However, most respondents viewed them as a treatment option rather than a treatment of choice with less than one-thirds using them when they were unable to provide a conventional restoration. It is not clear why more paediatric dentists and DwSI do not consider MID as the treatment of choice, given, the emergence of increasing evidence supporting a high success rate, acceptance among children and carers as well as ease of doing them compared to conventional restorations requiring local anaesthesia, placement of rubber dam and drilling.

Based on the findings of a recent published umbrella review on MID, there is a clear need to increase the emphasis on considering these techniques for managing carious primary teeth as a mainstream option rather than a compromise option in circumstances where the conventional approach is not possible due to cooperation or cost (BaniHani et al. 2021).

In addition, it is relevant to note that among the reasons listed for using MID techniques were an appropriate management technique for anxious children (79.2%) and young ones (74.5%). A finding that was confirmed by the majority of the respondents when they stated that their choice of using MID was affected by the child`s behaviour and cooperation. This is unsurprising given the fact that MID is considered a child-friendly approach where carious lesion could be either left (SDF), sealed (fissure sealants, resin infiltration and HT) or removed using hand instruments mainly (selective caries removal, stepwise and ART) without the need for local anaesthesia, rubber dam, or drilling. Thus less fear inducing for the young and anxious children compared to the conventional restoration.

COVID-19 has changed paediatric dentists and DwSI views and practices of MID, reported by more than one-third of the respondents. Practitioners that have practiced paediatric dentistry for over 15 years were less inclined to use MID post-COVID-19. This can likely be explained by the fact that these practitioners were already using MID rather intensively. Indeed, 61.11% of respondents reporting to have “Always” used MID pre-COVID-19 belonged to practitioners of over 15 years.

The survey highlighted many favourable comments mainly concerning the advantage of using MID in the management of carious primary teeth as LRAGPs, thus reducing the need for AGPs during the pandemic as well as the patient waiting lists for dental treatment, both in the chair and under general anaesthesia. Another interesting finding highlighted by the respondents was the emergence of the use of 38% SDF for the management of carious primary teeth in children in the U.K. Although SDF is not a new caries management technique with the literature reporting its first uses in Japan and China in the seventies and eighties, respectively, at concentrations varying from 10 to 38% to promote dental caries arrest, SDF gained a significant popularity among the paediatric dentists in the U.K during the pandemic (Seifo et al. 2020). Perhaps this is due to the simple application of SDF with no caries excavation being required prior to its application (Gao et al. 2021). In addition, the BSPD has published several SDF resources for dental caries management on their website for healthcare professionals, encouraging paediatric dentists and GDPs in the U.K to use it in children (BSPD 2020).

It emerged that, although very few in number, there are still some paediatric dentists and DwSI who are reluctant to adopt MID after the COVID-19 pandemic. The main reason for non-use was given as lack of teaching and training, lack of confidence, difficulty in obtaining dental materials required for MID along with a lack of confidence in the available evidence. These barriers were reported more by the respondents practicing in E.U, although the majority of these participants were clinical academics (30.3%), highlighting a need for supportive education and training on the use of different MID techniques including HT and SDF.

This study is not without limitations. Only members of the BSPD and EAPD were approached to participate in the study with the majority of the participants practicing in E.U followed by U.K with only 16% were based in non-E.U countries, therefore, generalisation of the results may not be appropriate. In addition, some of the BSPD members are also members of the EAPD causing possible overlap between the participants’ responses, it was not possible to identify and remove duplicates. However, participants who were members of both EAPD and BSPD were asked to complete the questionnaire once only. Moreover, the questionnaire was only available in the English language which can be a limitation for some of the EAPD members who do not speak the English language. Another limitation is that at the time of conducting the study in July 2021, it was still considered a COVID period in the U.K and most E.U countries, possibly impacting the results of the study.

## Conclusion

Several MID techniques are practiced broadly by a sample of paediatric dentists and DwSI in paediatric dentistry across the E.U and U.K During the COVID-19 pandemic, HT and topical application of 38% SDF were more widely used among the participants in the U.K, whereas the conventional restoration of non-selective caries removal and pulp therapy remained the most preferred technique for the management of carious primary teeth among the participants in the E.U. The pandemic served as an opportunity for many dentists to become familiar with various MID practices, such as SDF, which has been already established some time ago. It will be interesting to know if these practices remain in the armamentarium of the dental practice after a normalization is established. A small number of the paediatric dentists and DwSI, mainly in the E.U, are reluctant to adopt MID after the COVID-19 pandemic. Barriers such as lack of teaching and confidence, difficulty obtaining materials required for MID as well as perceived lack of evidence on the efficacy of MID were identified.

## References

[CR1] Alamoudi RA, Basudan S, Mahboub M, Baghlaf K (2022). Impact of COVID-19 pandemic on dental treatment in children: a retrospective cross-sectional analysis in Jeddah city. Clin Cosmet Investig Dent.

[CR2] Al-Halabi M, Salami A, Alnuaimi E (2020). Assessment of paediatric dental guidelines and caries management alternatives in the post COVID-19 period. A critical review and clinical recommendations. Eur Arch Paediatr Dent.

[CR3] BaniHani A, Deery C, Toumba J (2018). The impact of dental caries and its treatment by conventional or biological approaches on the oral health-related quality of life of children and carers. Int J Paediatr Dent.

[CR4] BaniHani A, Duggal M, Toumba J, Deery C (2018). Outcomes of the conventional and biological treatment approaches for the management of caries in the primary dentition. Int J Paediatr Dent.

[CR5] BaniHani A, Deery C, Toumba J, Duggal M (2019). Effectiveness, costs and patient acceptance of a conventional and a biological treatment approach for carious primary teeth in children. Caries Res.

[CR6] BaniHani A, Gardener C, Raggio DP, Santamaría RM, Albadri S (2020). Could COVID-19 change the way we manage caries in primary teeth? Current implications on Paediatric Dentistry. Int J Paediatr Dent.

[CR7] BaniHani A, Santamaría RM, Hu S (2021). Minimal intervention dentistry for managing carious lesions into dentine in primary teeth: an umbrella review. Eur Arch Paediatr Dent.

[CR8] British Society of Paediatric Dentistry. New SDF resources. Accessed on the 7th of October 2021. https://www.bspd.co.uk/Professionals/Resources.

[CR9] Dawson AS, Makinson OF (1992). Dental treatment and dental health. Part 1 A review of studies in support of a philosophy of Minimum Intervention Dentistry. Aust Dent J.

[CR10] de Amorim RG, Frencken JE, Raggio DP (2018). Survival percentages of atraumatic restorative treatment (ART) restorations and sealants in posterior teeth: an updated systematic review and meta-analysis. Clin Oral Investig.

[CR11] Doméjean S, Ducamp R, Léger S, Holmgren C (2015). Resin infiltration of non-cavitated caries lesions: a systematic review. Med Princ Pract.

[CR12] Fux-Noy A, Mattar L, Shmueli A (2021). Oral health care delivery for children during COVID-19 pandemic—a retrospective study. Front Public Health.

[CR13] Gao SS, Zhao IS, Hiraishi N (2016). Clinical trials of silver diamine fluoride in arresting caries among children: a systematic review. JDR Clin Transl Res.

[CR14] Gao SS, Amarquaye G, Arrow P (2021). Global oral health policies and guidelines: using silver diamine fluoride for caries control. Front Oral Health.

[CR15] Hussein I, Al Halabi M, Kowash M (2020). Use of the Hall technique by specialist paediatric dentists: a global perspective. Br Dent J.

[CR16] Innes NP, Stirrups DR, Evans DJ, Hall N, Leggate M (2006). A novel technique using preformed metal crowns for managing carious primary molars in general practice - a retrospective analysis. Br Dent J..

[CR17] Innes N, Stewart M, Souster G (2015). The Hall Technique; retrospective case-note follow-up of 5-year RCT. Br Dent J.

[CR18] Innes NP, Frencken JE, Bjørndal L (2016). Managing carious lesions: consensus recommendations on terminology. Adv Dent Res.

[CR19] Naaman R, El-Housseiny AA, Alamoudi N (2017). The use of pit and fissure sealants—a literature review. Dent J.

[CR20] RCPCH Research & Evidence team. COVID-19—research evidence summaries. Royal College of Paediatrics and Child Health. 2020. https://www.rcpch.ac.uk/resources/covid-19-research-evidence-summaries. Accessed on the 7th of Oct 2021.

[CR21] Roberts A, McKay A, Albadri S (2018). The use of Hall technique preformed metal crowns by specialist paediatric dentists in the UK. Br Dent J.

[CR22] Seifo N, Robertson M, MacLean J (2020). The use of silver diamine fluoride (SDF) in dental practice. Br Dent J.

[CR23] Tolba ZO, Hamza HS, Moheb DM (2019). Effectiveness of two concentrations 12% versus 38% of silver diamine fluoride in arresting cavitated dentin caries among children: a systematic review. Egypt Pediatr Assoc Gaz.

